# Temporal and geographic trends in mortality involving co-occurring depression and diabetes mellitus in U. S., 1999–2023

**DOI:** 10.3389/fmed.2026.1734188

**Published:** 2026-02-24

**Authors:** Xinyu Wang, Wenbin Xu, Zhinan Ye

**Affiliations:** 1 Department of Internal Medicine, Baotou Mongolian Medicine and Traditional Chinese Medicine Hospital, Baotou, China; 2 Department of Rehabilitation Medicine, Taizhou Municipal Hospital (Taizhou University Affiliated Municipal Hospital), School of Medicine, Taizhou University, Zhejiang, China; 3 Department of Neurology, Taizhou Municipal Hospital (Taizhou University Affiliated Municipal Hospital), School of Medicine, Taizhou University, Taizhou, Zhejiang, China

**Keywords:** age-adjusted mortality, CDC WONDER, depression, diabetes mellitus, geographic trends

## Abstract

**Background:**

Diabetes mellitus (DM) and depression commonly co-occur, worsening self-management, complications, and survival. Yet long-term, population-based surveillance of mortality that involves both conditions remains limited.

**Objective:**

To characterize national trends in mortality co-listing diabetes and depression and to examine disparities by demographics and geography.

**Methods:**

We analyzed U. S. Multiple Cause-of-Death records from CDC WONDER for 1999–2023. Deaths that listed diabetes (ICD-10 E10–E14) and depressive disorders (ICD-10 F32–F33, F41.2) as underlying or contributing causes were included. We calculated crude and age-adjusted mortality rates (AAMRs) per 100,000, directly standardized to the 2000 U. S. population, and fitted segmented Joinpoint regression to estimate annual percent change (APC) and average APC. Stratifications included sex, age group, race/ethnicity, Census region, state, urban–rural category (2020 NCHS scheme applied uniformly across years), and place of death, with adherence to CDC WONDER suppression rules.

**Results:**

National AAMRs were low and broadly stable through the mid-to-late 2000s, rose in the late 2010s, and plateaued in the early 2020s. Rates were consistently higher in men than women and increased with age, peaking in adults aged 85 years or older. By race/ethnicity, non-Hispanic American Indian/Alaska Native populations had the highest AAMRs, followed by non-Hispanic Black and Hispanic groups, with non-Hispanic White and non-Hispanic Asian/Pacific Islander populations lower. Regionally, the West had the highest rates and the Northeast the lowest; nonmetropolitan counties exceeded metropolitan counties. States showed wide heterogeneity, with roughly threefold differences between the top and bottom deciles.

**Conclusion:**

Mortality involving co-occurring depression and diabetes shows an upswing in the late 2010s and substantial demographic and geographic inequalities. These findings support targeted, place-based strategies and integrated diabetes–mental health care to reduce preventable deaths.

## 1 Introduction

Diabetes mellitus (DM) has increased in prevalence over recent decades and now constitutes a major global public-health burden (1, 2). In 2021, an estimated 529 million people were living with diabetes worldwide, and the age-standardized prevalence was 6.1%, up 90.5% from 1990 (3). Type 2 diabetes accounts for >96% of all diabetes cases. At the population level, high BMI contributed to >50% of global type 2 diabetes DALYs in 2021, underscoring adiposity as a central driver of risk. Prevalence is higher in males than females (male-to-female ratio 1.14 in 2021), with regional variation (4). Prevalence rises steeply with age, peaking at 24.4% in those 75–79 years and remaining <1% under 20 years. Beyond adiposity, diet, environmental/occupational exposures, tobacco use, low physical activity, and alcohol materially contribute to type 2 diabetes risk. As people with diabetes live longer, the profile of complications is shifting—traditional vascular outcomes now account for a smaller share of deaths, while “emerging” complications (including affective disorders) are increasingly recognized—reinforcing the need for sustained surveillance (5, 6).

As one of the most prevalent affective disorders, clinically significant depression is present in about one in four adults with type 2 diabetes, and the risk of major depression is roughly twice that seen in the general population (7). Meta-analyses and large cross-sectional datasets consistently show a substantially higher prevalence of depression in patients with diabetes, with estimates around 60% higher in individuals with type 2 diabetes and even larger elevations in some clinical settings (8). Co-occurring depression worsens diabetes self-management and glycaemic control, as patients are less likely to adhere to diet, physical activity, glucose monitoring, and medications. Additionally, pooled analyses link depression with higher average glucose levels (9). Depression is associated with medication non-adherence in type 2 diabetes, with downstream increases in both microvascular and macrovascular complications (10). Quantitatively, adults with diabetes who have depression experience about 50% higher all-cause mortality and about 40% higher cardiovascular mortality, alongside increased risks of coronary heart disease and stroke (11, 12).

The relationship between diabetes and depression is bidirectional: prior depression predicts incident type 2 diabetes across multiple longitudinal cohorts, and diabetes in turn elevates the subsequent risk of depression (13). Population syntheses reinforce this two-way linkage and document a rising co-prevalence over time. Shared pathophysiology spans hypothalamic–pituitary–adrenal axis hyperactivity with hypercortisolaemia, low-grade inflammation and cytokine signaling, insulin resistance in peripheral and central tissues, and gut–brain axis perturbations (14, 15). Neuroimaging and neuropathology point to hippocampal atrophy and altered large-scale connectivity as neural substrates linking metabolic dysregulation to mood disturbance (16).

Prior work has concentrated on mechanistic pathways, advancing understanding of the diabetes–depression nexus. Yet a translational gap persists between mechanistic evidence and population-level risk profiling, and systematic, comparable data on heterogeneity by sex, age, race/ethnicity, urban–rural residence, and region or state remain scarce. Our team’s literature review further indicates that recent nationwide studies often treat diabetes and mental disorders as a broad aggregate rather than isolating outcomes in which depression and diabetes co-occur, potentially overlooking features unique to this comorbidity (17). These gaps underscore the need for a reproducible, comparable, nationwide surveillance framework to characterize risks and inform policy.

To fill this gap, we present a national, population-based analysis of depression-associated mortality in the U. S. diabetes population (1999–2023), defining outcomes from death certificates, estimating age-adjusted mortality rates and APC/AAPC via Joinpoint regression, and evaluating heterogeneity across sex, age, race/ethnicity, urban–rural residence, region, and state. This framework addresses the surveillance gap and informs integrated diabetes–mental-health policy.

## 2 Methods

### 2.1 Data source and study population

We conducted a nationwide, population-based time-trend study using the publicly available Multiple Cause-of-Death (MCOD) files accessed via CDC WONDER. These files contain de-identified U. S. resident death certificates, with causes of death coded under the International Statistical Classification of Diseases and Related Health Problems, Tenth Revision (ICD-10). In this study, decedents with diabetes were identified using ICD-10 codes E10–E14; depressive disorders were ascertained using ICD-10 codes F32 (major depressive episode), F33 (recurrent depressive disorder), and F41.2 (mixed anxiety and depressive disorder). Because the dataset is publicly available and fully de-identified, institutional review board oversight was not required.

### 2.2 Study population and period

We extracted year, population denominators, and covariates including sex, race/ethnicity, age, urban–rural status, U. S. Census region, state of residence, and place of death. Diabetes mellitus was identified using ICD-10 codes E10–E14. Given that death certificates frequently lack specific diabetes typing (often defaulting to E14 “Unspecified diabetes mellitus”) and that mortality counts in the youngest age bands were extremely low, we analyzed the total diabetes burden without stratifying by type 1 versus type 2 diabetes. Race/ethnicity followed CDC WONDER conventions and was classified as non-Hispanic (NH) White, NH Black or African American, Hispanic/Latino, and NH other races. Age was grouped into nine bands (5–14, 15–24, 25–34, 35–44, 45–54, 55–64, 65–74, 75–84, and ≥85 years). Urban–rural status was assigned using the 2023 National Center for Health Statistics Urban–Rural Classification Scheme for Counties and summarized as metropolitan versus nonmetropolitan areas, and Census regions were defined as Northeast, Midwest, South, and West. Place of death was coded per CDC WONDER as medical facility (hospital outpatient/emergency department, hospital inpatient, dead on arrival), nursing home/long-term care facility, hospice facility, home, or other/unknown. All analyses adhered to CDC WONDER small-number rules: cell counts from 0 to 9 were suppressed and rates based on fewer than 20 deaths were flagged as statistically unreliable; strata affected by suppression or unreliability were excluded from choropleth mapping and trend modeling.

### 2.3 Sensitivity analyses

We computed crude and age-adjusted mortality rates (AAMRs) per 100,000 persons annually for 1999–2023. Crude rates were defined as the number of deaths meeting the case definition divided by the corresponding U. S. population for that year; AAMRs were derived by direct standardization to the 2000 U. S. standard population, with 95% confidence intervals. Temporal trends were assessed using the National Cancer Institute’s Joinpoint Regression Program, which fits log-linear segmented models to estimate segment-specific annual percent change (APC) and average annual percent change (AAPC), each with 95% confidence intervals. APCs were classified as increasing or decreasing when the model slope differed significantly from zero on two-sided tests (*α* = 0.05); to limit overfitting, models were constrained to no more than three joinpoints (minimum segment length ≥3 years), and permutation procedures (or information criteria, as appropriate) informed model selection. To stabilize sparse strata, we applied 3-year moving averages and adhered to CDC WONDER small-number rules (suppressing counts of 0–9 and flagging rates based on <20 deaths as statistically unreliable); such strata were excluded from mapping and trend models but retained in national aggregates. Data were extracted in CDC WONDER; rate construction and graphics were performed in R (version 4.5.1); segmented trend models were fit in the NCI Joinpoint Regression Program (version 4.0.0).

## 3 Results

**Figure 1:**
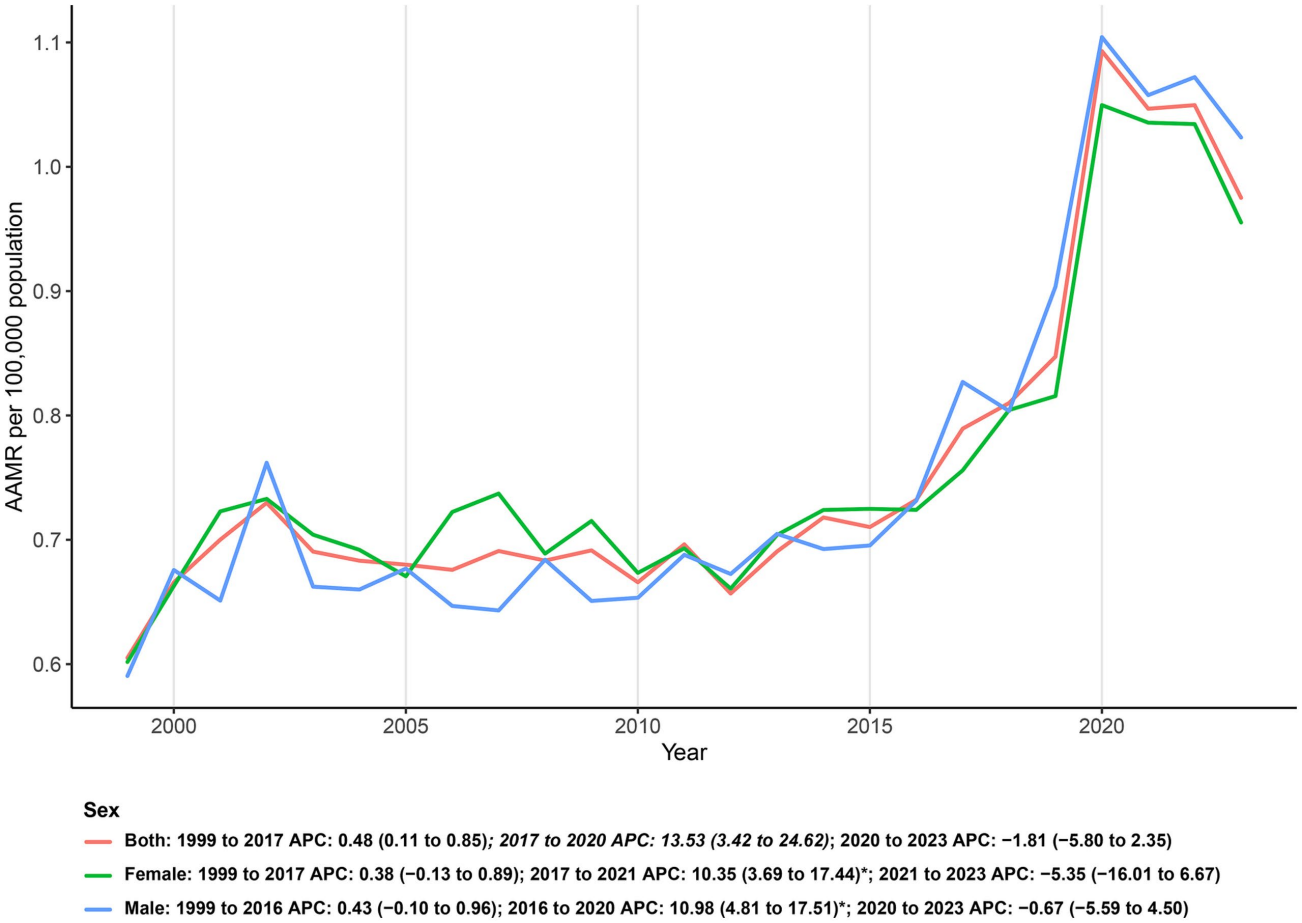
Sex-stratified age-adjusted mortality rates (per 100,000) for co-listed diabetes and depression in U. S., 1999–2023.

From 1999 through 2023, a total of 62,593 deaths were identified among U. S. adults with DM and depression co-listed on the death certificate, corresponding to an overall age-adjusted mortality rate of 0.76 per 100,000 (95% CI: 0.73 to 0.79). Over the full period, the AAMR increased on average (AAPC: 1.73; 95% CI: 0.50 to 2.97). Joinpoint regression indicated three phases: the rate was largely stable during 1999–2017 (APC: 0.48; 95% CI: 0.11 to 0.85), rose significantly in 2017–2020 (APC: 13.53; 95% CI: 3.42 to 24.62), and showed no significant change in 2020–2023 (APC: −1.81; 95% CI: −5.80 to 2.35). This pattern reflects an extended period of minimal change followed by a sharp late-2010s increase and a subsequent plateau in the early 2020s (Figure 1; Table 1).

**Table 1:** Trends in deaths and age-adjusted mortality for deaths co-mentioning DM and depression, United States, 1999–2023.

Characteristic	Deaths			AAMR		
	1999	2023	Percent Change (%)	1999 (95% CI)	2023 (95% CI)	AAPC (95% CI)
Total	1511	3924	159.70	0.61 (0.57 to 0.64)	0.98 (0.94 to 1.01)	1.73 (0.50 to 2.97)*
Sex						
Female	919	2091	127.53	0.60 (0.56 to 0.64)	0.96 (0.91 to 1.00)	1.48 (0.09 to 2.89)*
Male	592	1833	209.63	0.59 (0.54 to 0.64)	1.02 (0.98 to 1.07)	1.98 (0.84 to 3.12)*
Census region						
Northeast	285	608	113.33	0.53 (0.47 to 0.60)	0.82 (0.75 to 0.88)	1.25 (−0.92 to 3.47)
Midwest	550	950	72.73	0.91 (0.83 to 0.99)	1.13 (1.06 to 1.20)	1.09 (−0.81 to 3.02)
South	420	1509	259.29	0.47 (0.43 to 0.52)	1.01 (0.96 to 1.06)	3.01 (1.77 to 4.26)*
West	256	857	234.77	0.50 (0.43 to 0.56)	0.95 (0.89 to 1.02)	3.14 (1.62 to 4.69)*
Race						
Hispanic	61	320	424.59	0.51 (0.38 to 0.66)	0.78 (0.69 to 0.86)	2.89 (2.14 to 3.65)*
NH Black	130	356	173.85	0.58 (0.48 to 0.69)	0.87 (0.77 to 0.96)	2.37 (1.44 to 3.31)*
NH White	1302	3123	139.86	0.60 (0.57 to 0.63)	1.10 (1.06 to 1.14)	2.49 (0.87 to 4.14)*
NH Other	13	96	638.46	NA	0.40 (0.33 to 0.49)	2.02 (0.84 to 3.20)*
Urbanization^1^						
Metropolitan	1144	3218	181.29	0.57 (0.54 to 0.60)	0.99 (0.95 to 1.02)	2.58 (1.33 to 3.83)*
Nonmetropolitan	367	943	156.95	0.76 (0.68 to 0.84)	1.51 (1.41 to 1.61)	2.42 (1.45 to 3.40)*
Age Groups^2^						
35–44 years	26	64	146.15	0.06 (0.04 to 0.08)	0.14 (0.11 to 0.18)	3.91 (0.19 to 7.77)*
45–54 years	75	217	189.33	0.21 (0.16 to 0.26)	0.54 (0.46 to 0.61)	3.81 (2.02 to 5.64)*
55–64 years	134	556	314.93	0.56 (0.47 to 0.66)	1.33 (1.22 to 1.44)	3.49 (3.06 to 3.92)*
65–74 years	323	1002	210.22	1.75 (1.56 to 1.94)	2.89 (2.71 to 3.07)	2.29 (1.20 to 3.39)*
75–84 years	574	1195	108.19	4.70 (4.31 to 5.08)	6.51 (6.14 to 6.87)	1.44 (0.71 to 2.18)*
85 + years	371	857	131.00	8.93 (8.02 to 9.84)	13.83 (12.91 to 14.76)	1.40 (0.68 to 2.13)*

AAMR, age-adjusted mortality rate; AAPC, average annual percent change; CI, confidence interval; DM, diabetes mellitus; NH, non-Hispanic.

^*^Statistically significant (*p* < 0.05).

^1^For urbanization, the 2023 AAMR was substituted with the 2020 value, and the AAPC was calculated for 1999–2020.

^2^For age groups, AAMRs were replaced with crude mortality rates, and the AAPCs were derived accordingly.

### 3.1 Gender trend

Across the study period, AAMRs were generally higher in males than in females, with the gap most apparent in recent years. In 1999, AAMRs were similar between sexes, with rates of 0.59 (95% CI: 0.54 to 0.64) for males and 0.60 (95% CI: 0.56 to 0.64) for females; however, by 2023, males exceeded females, reaching 1.02 (95% CI: 0.98 to 1.07) compared to 0.96 (95% CI: 0.91 to 1.00). Joinpoint analysis showed no significant change for males during 1999–2016 (APC: 0.43; 95% CI: −0.10 to 0.96), followed by a significant increase in 2016–2020 (APC: 10.98; 95% CI: 4.81 to 17.51) and no significant change in 2020–2023 (APC: −0.67; 95% CI: −5.59 to 4.50), yielding an overall AAPC: 1.98 (95% CI: 0.84 to 3.12). For females, the pattern was similar: no significant change in 1999–2017 (APC: 0.38; 95% CI: −0.13 to 0.89), a significant increase in 2017–2021 (APC: 10.35; 95% CI: 3.69 to 17.44), and no significant change in 2021–2023 (APC: −5.35; 95% CI: −16.01 to 6.67), with an overall AAPC: 1.48 (95% CI: 0.09 to 2.89). Collectively, both sexes exhibited a prolonged period of minimal change, a pronounced rise in the late 2010s, and a subsequent plateau in the early 2020s, with levels and growth modestly higher in males (Figure 1; Supplementary Table 1).

### 3.2 Census region

**Figure 2:**
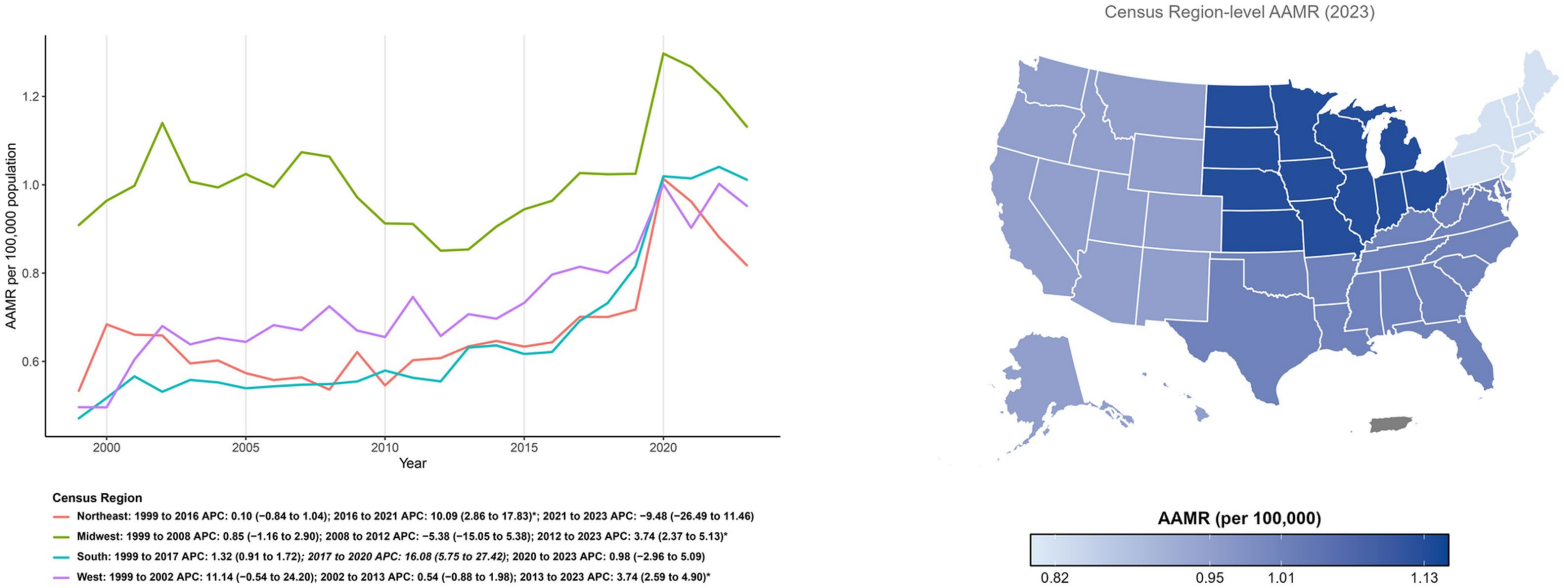
Temporal trends and geographic distribution of age-adjusted mortality (per 100,000) involving co-occurring diabetes and depression by U. S. Census Region, 1999–2023. The main line graph illustrates the annual trends in age-adjusted mortality rates (AAMR) for the Northeast, Midwest, South, and West regions over the study period. The inset map visualizes the geographic variation in mortality rates in 2023, where darker shading signifies a higher mortality burden (Midwest > South > West > Northeast).

Geographic variations in mortality are visually presented in Figure 2. As illustrated by the region map, the Midwest exhibited the highest burden in 2023 (AAMR: 1.13 per 100,000), followed by the South (1.01) and West (0.95), with the Northeast showing the lowest rates (0.82). Longitudinally, AAMRs varied by Census region and were consistently highest in the Midwest across the study period. In 1999, the lowest rates were observed in the South, whereas by 2023, the Northeast had the lowest rates. Joinpoint analyses identified region-specific accelerations in the late 2010s: the South increased during 2017–2020 (APC: 16.08; 95% CI: 5.75 to 27.42); the West rose steadily across 2013–2023 (APC: 3.74; 95% CI: 2.59 to 4.90); the Northeast increased in 2016–2021 (APC: 10.09; 95% CI: 2.86 to 17.83) and then showed no significant change in 2021–2023 (APC: −9.48; 95% CI: −26.49 to 11.46); and the Midwest increased in 2012–2023 (APC: 3.74; 95% CI: 2.37 to 5.13) (Figure 2; Supplementary Table 2).

### 3.3 Ethnicity trend

Over 1999–2023, AAMRs differed across race/ethnicity, with the highest 2023 level in NH White (1.10; 95% CI: 1.06 to 1.14), followed by NH Black (0.87; 95% CI: 0.77 to 0.96), Hispanic (0.78; 95% CI: 0.69 to 0.86), and NH Other (0.40; 95% CI: 0.33 to 0.49). Compared with 1999, AAMRs rose in NH White (0.60 to 1.10) and NH Black (0.58 to 0.87); Hispanic also increased (0.51 to 0.78), while the earliest AAMR for NH Other was unstable due to small numbers. Cumulatively, deaths were most numerous among NH White (*n* = 51,396), with smaller totals in NH Black (*n* = 5,077), Hispanic (*n* = 4,360), and NH Other (*n* = 1,563).

**Figure 3:**
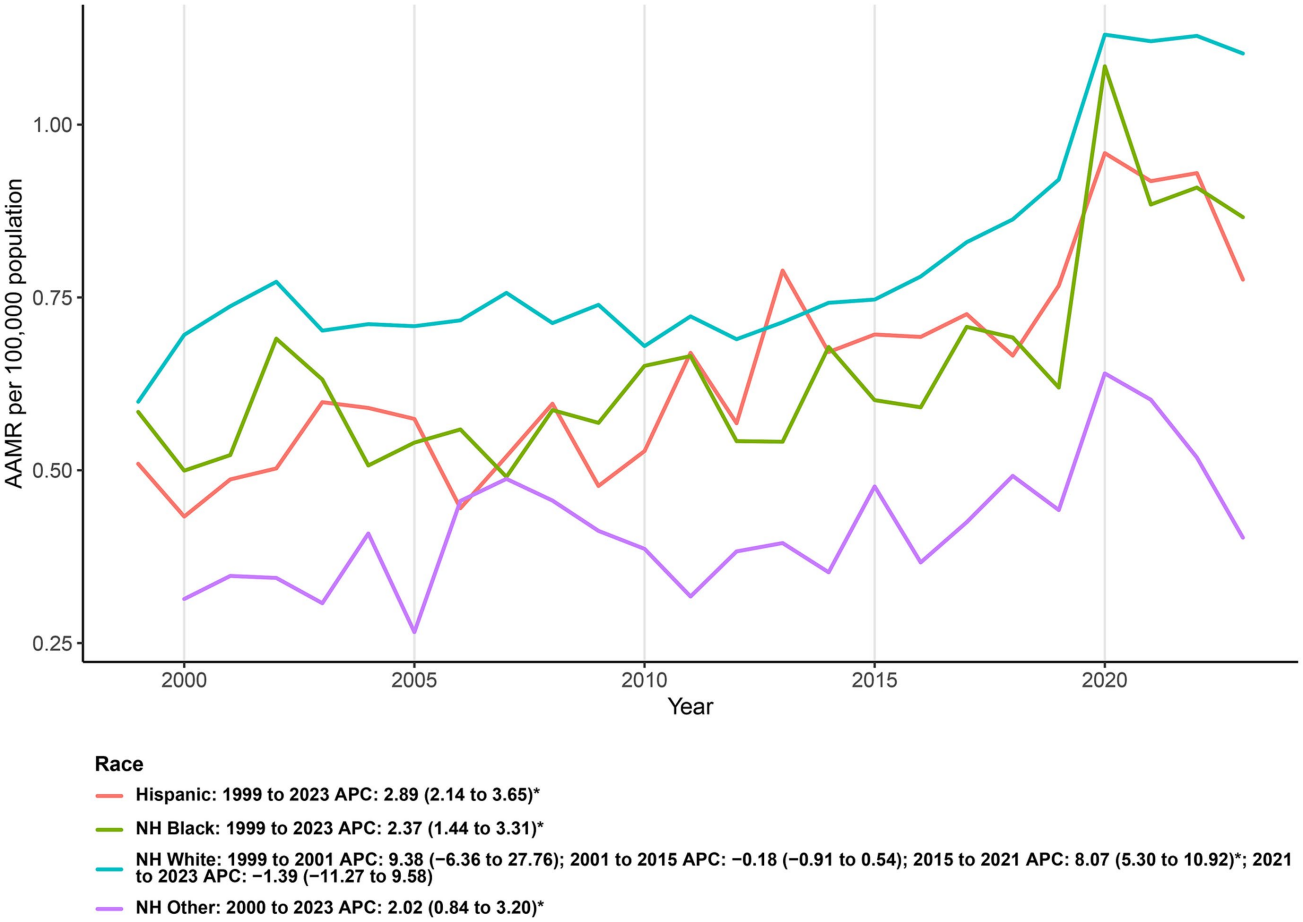
Ethnicity-stratified age-adjusted mortality rates (per 100,000) for deaths co-listing diabetes and depression, United States, 1999–2023.

Joinpoint results indicated monotonic increases for Hispanic (AAPC: 2.89; 95% CI: 2.14 to 3.65), NH Black (AAPC: 2.37; 95% CI: 1.44 to 3.31), and NH Other (AAPC: 2.02; 95% CI: 0.84 to 3.20). In contrast, NH White exhibited a multiphase pattern: a non-significant change in 2001–2015 (APC: −0.18; 95% CI: −0.91 to 0.54), followed by a significant increase in 2015–2021 (APC: 8.07; 95% CI: 5.30 to 10.92) and no significant change in 2021–2023 (APC: −1.39; 95% CI: −11.27 to 9.58). Collectively, these findings show sustained upward trends across all groups, with the highest current rates in NH White and pronounced late-2010s acceleration most evident in NH White (Figure 3; Supplementary Table 3).

### 3.4 Age group trends

**Figure 4:**
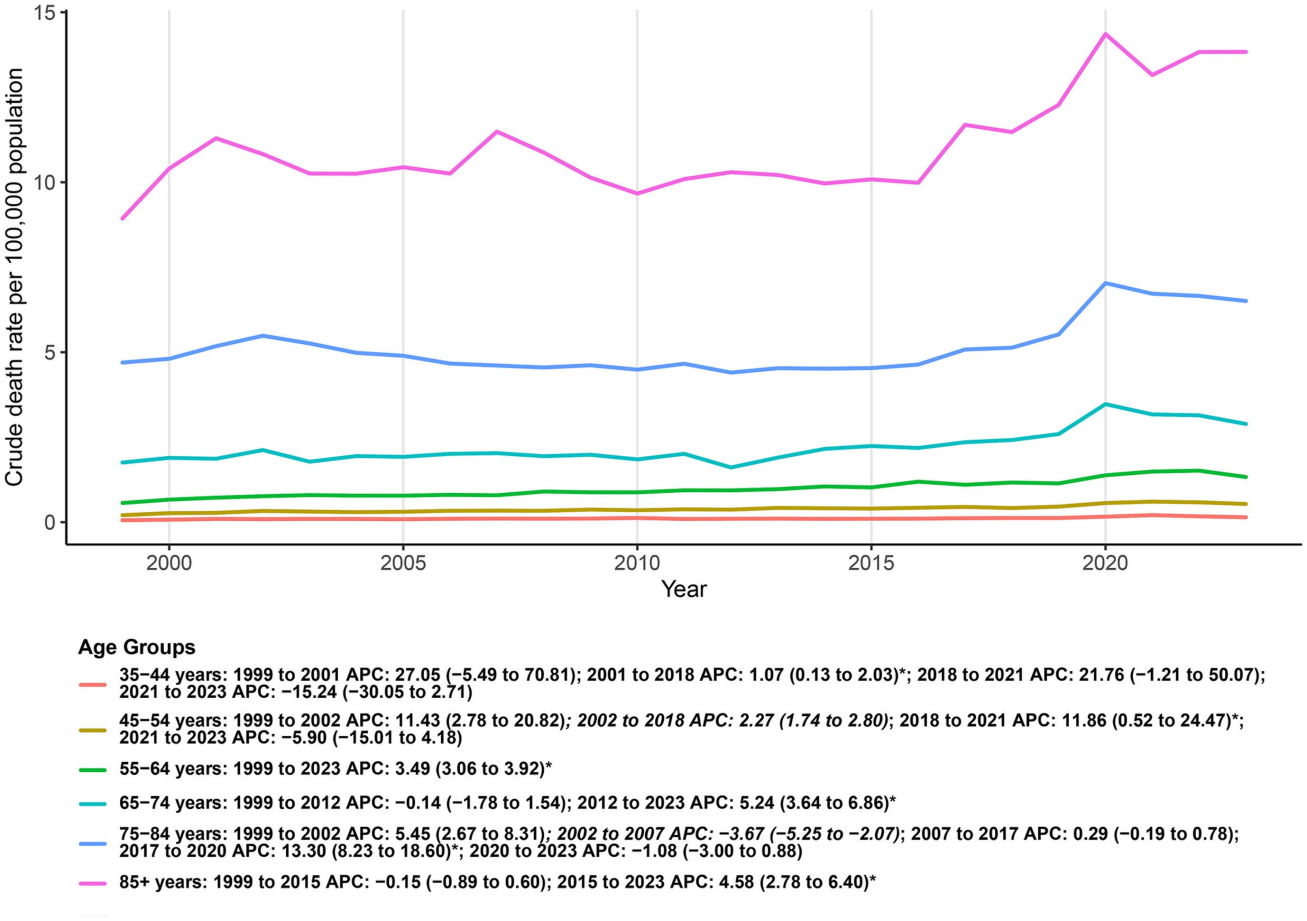
Age-group patterns in crude mortality (per 100,000) for deaths co-listing diabetes and depression among U. S. adults aged ≥35 years, 1999–2023.

Because cell counts were sparse and frequently suppressed under CDC WONDER small-number rules, the three youngest strata (5–14, 15–24, and 25–34 years) were excluded from trend modeling; descriptive summaries are provided in the Supplement. Among included age groups (35–44, 45–54, 55–64, 65–74, 75–84, and ≥85 years), AAMRs displayed a clear age gradient, remaining lowest in 35–44 years and highest in ≥85 years throughout the study period. Joinpoint analyses revealed broadly concordant temporal patterns across age bands: a prolonged phase of little or no change through the early–mid 2010s, a marked upswing in the late 2010s, and no significant change thereafter in the early 2020s. The late-2010s acceleration was most pronounced in 65–74, 75–84, and ≥85 years, more modest in 35–44 and 45–54 years, and intermediate in 55–64 years. Taken together, these findings indicate that the recent increase in DM–depression co-listed mortality was concentrated among older adults, whereas younger middle-aged groups experienced lower absolute levels and more muted changes (Figure 4; Supplementary Table 4).

### 3.5 Urbanization trends

**Figure 5:**
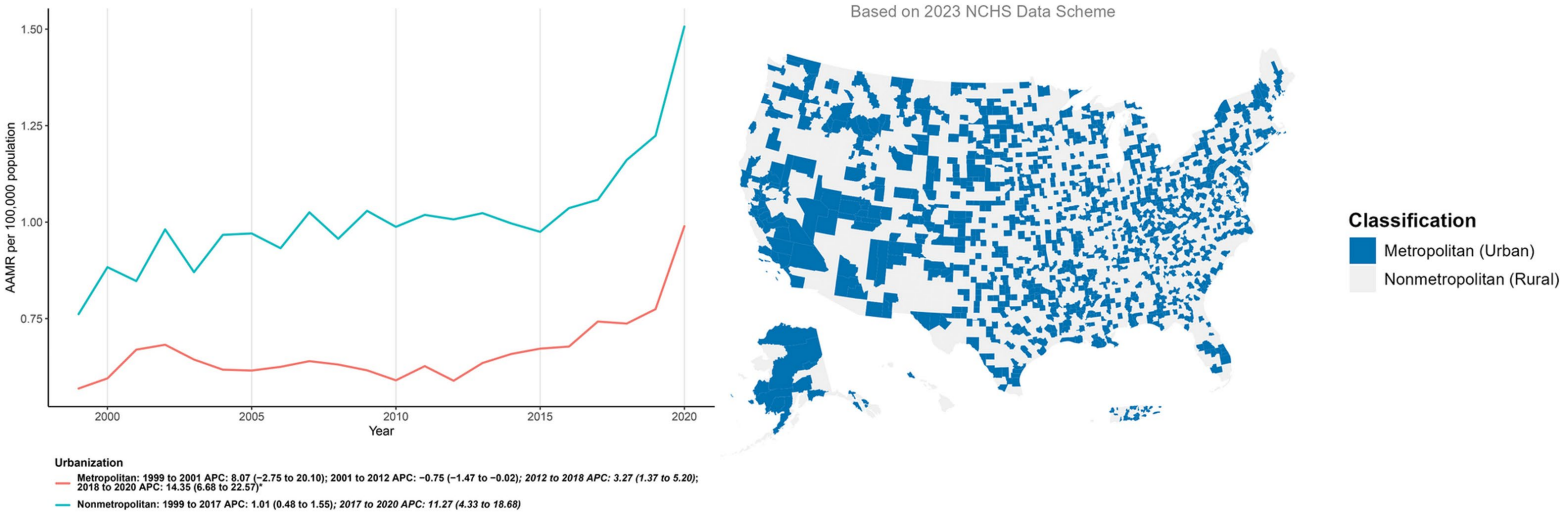
Temporal trends and geographic distribution of age-adjusted mortality (per 100,000) involving co-occurring diabetes and depression by urbanization level, United States, 1999–2023. The main line graph illustrates the annual trends in age-adjusted mortality rates (AAMR) stratified by metropolitan and nonmetropolitan areas over the study period. The inset map visualizes the geographic classification of U. S. counties based on the 2023 National Center for Health Statistics (NCHS) Urban–Rural Classification Scheme for Counties, distinguishing between metropolitan (codes 1–4) and nonmetropolitan (codes 5–6) areas.

The geographic distribution of metropolitan and nonmetropolitan counties is visually presented in Figure 5. Consistent with this classification, AAMRs were consistently higher in nonmetropolitan than in metropolitan counties. Metropolitan counties declined modestly in 2001–2012 (APC: −0.75; 95% CI: −1.47 to −0.02), then increased in 2012–2018 (APC: 3.27; 95% CI: 1.37 to 5.20) and accelerated in 2018–2020 (APC: 14.35; 95% CI: 6.68 to 22.57). Nonmetropolitan areas rose gradually in 1999–2017 (APC: 1.01; 95% CI: 0.48 to 1.55) with a sharper uptick in 2017–2020 (APC: 11.27; 95% CI: 4.33 to 18.68). Urbanization status was assigned using the 2023 NCHS county classification and applied uniformly across all study years (Figure 5; Supplementary Table 5).

### 3.6 Geography trends

State-level heterogeneity was pronounced. Jurisdictions in the 90th percentile of AAMRs (Vermont, Oregon, North Dakota, Nebraska, and Kentucky) had per 100,000 rates nearly three times those in the 10th percentile (Florida, Alabama, California, Massachusetts, and Nevada) (Supplementary Table 6).

## 4 Discussion

Using national death-certificate data from 1999 to 2023, we observed a long period of minimal change in DM–depression co-listed mortality, a discrete upswing in the late 2010s, and stabilization in the early 2020s. Men consistently had higher age-adjusted mortality than women, and rates rose steeply with age, peaking in those aged ≥85 years. By race/ethnicity, recent levels were highest in non-Hispanic White adults, with sustained increases across groups that differed in timing and magnitude. Geographically, the West had the highest rates, the Midwest and South were intermediate, and the Northeast was lowest. After standardizing urbanization to the 2020 NCHS scheme, nonmetropolitan counties maintained higher rates than metropolitan counties. State-level dispersion was wide, with jurisdictions in the upper decile approaching threefold the rates in the lowest decile. Most deaths occurred in medical facilities, followed by home and hospice settings. Taken together, these patterns delineate the national burden and highlight populations and places where integrated diabetes–mental health prevention and care may yield the greatest benefit.

We observed an inflection in mortality coincident with the onset of the COVID-19 pandemic, with a long-standing plateau giving way to a short-lived upswing and subsequent stabilization; this pattern may reflect pandemic-related disruptions in care and heightened psychological distress. Several interacting mechanisms may account for this pattern. Disruptions in healthcare access, including reduced availability of routine follow-up, medications, and laboratory testing, likely impaired metabolic control in diabetic patients (18, 19). Psychological distress driven by isolation, uncertainty, and fear may have intensified depressive symptoms, which are known to worsen diabetes outcomes (20). In addition, persistent post-infection symptoms, chronic inflammation, and microvascular dysfunction associated with long COVID appeared to affect diabetic individuals more frequently (21). These temporally aligned factors suggest the need to strengthen continuity of chronic disease management, mental health services, and integrative care for post-COVID complications in future public health emergencies.

In sex-stratified analyses, AAMRs were consistently higher in males than in females throughout the study period. Both sexes showed a prolonged interval of minimal change, a distinct upswing in the late 2010s, and stabilization in the early 2020s. This temporal pattern was broadly concordant in national aggregates and across major subgroups defined by region, urbanization, and race or ethnicity. The ordering of males and females remained stable over time and was evident in most age groups. Year-to-year fluctuations were modest relative to the overall trajectory and did not alter the persistent male excess observed in the primary summaries. Sex differences are likely driven by a combination of biological and behavioral factors. Women with diabetes bear a higher and earlier-onset burden of depression across the life course, most pronounced in midlife, which may amplify risk through pathways involving treatment adherence and lifestyle behaviors (22). Men are comparatively disadvantaged with respect to atherosclerosis, microvascular endothelial dysfunction, prothrombotic states, and oxidative stress profiles; together with exposures such as smoking, these factors more readily culminate in fatal cardiovascular events (23). The interplay between depression and cardiovascular burden appears sex specific, and insufficient sleep can further exacerbate adverse outcomes, underscoring the need for differentiated preventive and therapeutic strategies in men and women (24).

Among adults with diabetes and co-listed depression, recent mortality levels were highest in non-Hispanic White individuals, followed by non-Hispanic Black and Hispanic groups, with non-Hispanic Other lowest. This finding stands in contrast to general surveillance data indicating that the burden of type 2 diabetes is disproportionately higher in non-White populations. The higher observed mortality in White individuals may reflect disparities in mental health diagnosis and reporting; specifically, greater access to mental health care among White populations can lead to higher rates of depression diagnosis and subsequent documentation on death certificates, whereas mood disorders may be underdiagnosed and underreported in minority groups due to systemic barriers. Despite these observational trends, structural inequities remain critical: national survey data indicate that adults with type 2 diabetes who identify as Black, American Indian or Alaska Native, or multiracial are more likely than White adults to lack reliable transportation for health-related needs, a barrier linked to gaps in care and glycemic control (25). Furthermore, beyond access barriers, US mortality analyses continue to show persistent racial differences in cardiometabolic outcomes among people with diabetes, with African American adults exhibiting higher age-adjusted mortality than White adults, reinforcing the need for equity-oriented strategies that address both healthcare access and community resources (26). These observations highlight the need for culturally responsive, integrated care pathways and improved surveillance that captures both mental-health and diabetes quality metrics across racial and ethnic groups.

In our data, age-specific gradients were pronounced: age-adjusted mortality increased stepwise across strata, with the oldest adults (≥85 years) bearing the heaviest burden. This pattern is compatible with evidence from late-life cohorts showing that clinically significant depression in older adults is associated with higher subsequent risk of diabetes, reinforcing the vulnerability of advanced age when mood disorder and diabetes intersect (27–29). In addition, biological changes that accompany aging, including reproductive and endocrine transitions, have been linked to greater incidence of type 2 diabetes, supporting the premise that age-related metabolic susceptibility can amplify mortality when depression and diabetes co-occur (30, 31).

At the Census region and urbanization level, we found that from 1999 to 2023 AAMRs were highest in the West and lowest in the Northeast, with late-2010s accelerations across regions; nonmetropolitan counties consistently exceeded metropolitan counties. At the state level, we found pronounced dispersion, with 90th-percentile states recording nearly three times the age-adjusted mortality of 10th-percentile states. Regional patterns in mortality involving co-occurring depression and diabetes likely reflect the geography of social risk and the organization of chronic-care services (32). Urbanization is consistently associated with higher diabetes prevalence than rural residence and with older age, expanding the pool at risk for comorbidity in more urbanized or aging regions (33). Policy implications from this evidence emphasize strengthening health-system capacity, improving access through universal coverage reforms, and maintaining robust NCD surveillance in both urban and rural cohorts to guide service planning and integration (34, 35).

Although this study provides a nationwide overview of mortality involving co-occurring depression and DM in the United States, several limitations remain. First, case ascertainment relies on death certificates; depression and other mental health conditions are often under-recorded, which can introduce misclassification and bias estimates downward. Second, we were unable to distinguish between type 1 and type 2 diabetes due to inherent limitations in death certificate coding, where a substantial proportion of deaths are assigned the unspecified code (ICD-10 E14) rather than specific types. Consequently, and given the negligible mortality counts observed in the youngest age groups (5–24 years), our findings predominantly reflect the epidemiological patterns of type 2 diabetes in older adults rather than type 1 diabetes. Third, the public-use files lack other clinical detail and socioeconomic context, preventing adjustment for depression severity, treatment exposure, glycemic control, multimorbidity, income, and related factors. Fourth, the observational, ecological design precludes causal interpretation of temporal patterns, and overlapping influences such as coding practices, changes in service availability, and macro-level shocks cannot be disentangled. Fifth, heterogeneity in certification practices across jurisdictions and the use of a single urban–rural classification applied uniformly across years, while improving comparability, may yield exposure misclassification when county characteristics evolve over time. Finally, when counts are small, particularly in younger age groups and at the state level, estimates become imprecise; missing or suppressed data can limit completeness and may attenuate or mask true differences. Accordingly, the findings should be interpreted with appropriate caution.

Future research should link death registry records with longitudinal clinical and claims data to calibrate depression severity, treatment trajectories, and glycemic control, and to elucidate potential pathways and competing risks. Prospective and quasi-experimental designs are needed to test whether collaborative care and other integrated models can reduce mortality among people with co-occurring depression and diabetes, including the evaluation of targeted mental health interventions in high-risk diabetes populations.

## 5 Conclusion

In this national, population-based analysis of U. S. mortality involving co-occurring depression and DM from 1999 to 2023, we document a long period of relative stability followed by an uptick in the late 2010s and a subsequent leveling in the early 2020s, with consistent gradients by sex, age, race and ethnicity, region, and urbanization. Males, older adults, and nonmetropolitan counties bore higher burdens; American Indian and Alaska Native communities and several western and midwestern jurisdictions were disproportionately affected, while the Northeast remained lowest. These patterns are concordant with independent CDC WONDER studies of depression-related mortality and highlight persistent geographic and demographic inequities. Methodologically, our use of age adjustment and Joinpoint regression provides a reproducible framework to quantify inflection points and compare trends across strata, complementing prior national surveillance of mental-health outcomes. The findings argue for integrated models that embed routine depression screening, treatment, and follow-up within diabetes care pathways, with priority for high-risk groups and rural settings where access constraints are greatest. Continued linkage of mortality surveillance with clinical and social data will be essential to refine risk attribution and to evaluate targeted prevention at scale.

## Data Availability

The datasets presented in this study can be found in online repositories. The names of the repository/repositories and accession number(s) can be found in the article/Supplementary material.
